# Carbyne Polysulfide as a Novel Cathode Material for Rechargeable Magnesium Batteries

**DOI:** 10.1155/2014/107918

**Published:** 2014-01-21

**Authors:** Yanna NuLi, Qiang Chen, Weikun Wang, Ying Wang, Jun Yang, Jiulin Wang

**Affiliations:** ^1^Department of Chemical Engineering, Shanghai Jiao Tong University, Shanghai 200240, China; ^2^Hirano Institute for Materials Innovation, Shanghai Jiao Tong University, Shanghai 200240, China; ^3^Research Institute of Chemical Defense, West Building, No. 35 Huayuanbei Road, Beijing 100083, China; ^4^Key Laboratory for Thin Film and Microfabrication of the Ministry of Education, Shanghai Jiao Tong University, Shanghai 200240, China

## Abstract

We report the formation of carbyne polysulfide by coheating carbon containing carbyne moieties and elemental sulfur. The product is proved to have a sp^2^ hybrid carbon skeleton with polysulfide attached on it. The electrochemical performance of carbyne polysulfide as a novel cathode material for rechargeable magnesium batteries is firstly investigated. The material exhibits a high discharge capacity of 327.7 mAh g^−1^ at 3.9 mA g^−1^. These studies show that carbyne polysulfide is a promising candidate as cathode material for rechargeable Mg batteries if the capacity retention can be significantly improved.

## 1. Introduction

It is well known that one of the greatest challenges of modern electrochemistry is the development of high energy density, rechargeable batteries for applications such as electric vehicles and large-scale power storage systems. Rechargeable magnesium battery system with magnesium anode may provide an alternative candidate that is competitive with post Li-ion batteries containing a lithium metal anode due to a relatively low price ($2700/ton and $64,800/ton for Mg and Li, resp.), a high theoretical volumetric capacity (3,832 mAh cm^−3^ for Mg and 2,062 mAh cm^−3^ for Li), and a higher expected safety (less dendritic morphologies for magnesium deposits than lithium) for magnesium compared with lithium [1, 2]. In 2000, a prototype with Mg_*x*_Mo_6_S_8_ (0 < *x* < 2) cathode and Mg(AlCl_2_EtBu)_2_/THF (Bu = butyl, Et = ethyl) electrolyte described by Aurbach et al. paves the way for the realization of rechargeable magnesium batteries [3]. Over the years, there have been continuous efforts in developing electrolyte systems with a wider electrochemical window and alternative cathode materials with a higher capacity and voltage plateau [4–12].

Compared with similarly sized and monovalent Li^+^ ion, Mg^2+^ ion has a stronger polarization due to the bivalence. A fundamental challenge of magnesium intercalation in inorganic hosts is the difficulty of Mg^2+^ insertion and the slow kinetics of Mg^2+^ diffusion [13, 14]. We proposed a class of energy storage materials, organosulfur compounds with S–S bonds, that is, 2,5-dimercapto-1,3,4-thiadiazole (DMcT), poly-2,2′dithiodianiline (PDTDA), and a conductive sulfur-containing material (CMS), as novel cathode materials for rechargeable magnesium batteries [15]. The capacity of the organosulfur compounds in 0.25 mol L^−1^ Mg(AlCl_2_EtBu)_2_/THF was low and decayed seriously probably resulting from the structure instability and the partial dissolution of discharge products in the electrolyte. A modified approach was to design some electrically conducting polymeric organosulfides, which keep conjugated backbones at both oxidation and reduction states and suitable sites of interpolymer disulfide bonds among different polymer chains. Herein, we prepared a novel carbyne polysulfide material containing a conducting carbon skeleton and energy-storing sulfur side chain without other elements by coheating carbyne and sulfur. Furthermore, the electrochemical performance of carbyne polysulfide as a novel cathode material for rechargeable magnesium batteries was investigated.

## 2. Experimental 

### 2.1. Preparation of Materials

Carbyne polysulfide material was synthesized by coheating carbyne and sulfur. 20 g polyvinylidene chloride (PVDC) was slowly added into the mixture of 800 mL saturated KOH ethanol solution (25 mass.%) and 1200 mL tetrahydrofuran (THF). After reacting under N_2_ atmosphere for 1 h at room temperature, the reaction liquid was neutralized with 2 mol L^−1^ HCl solution. Precipitate was collected by filtration and washed with distilled water and acetone for several times. Black carbyne powder was obtained after drying the precipitate under vacuum at room temperature. Then, the carbyne powder was mixed with sulfur at a molar ratio of 1 : 5 and ball-milled for 1 h. The mixture was heated at different temperatures (150°C, 200°C, 250°C, 300°C, and 350°C) at 10°C min^−1^ in a tube furnace, kept at the temperature for 3 h under a N_2_ atmosphere, and then allowed to cool to room temperature naturally. The products were marked as S1, S2, S3, S4, and S5, respectively.

### 2.2. Preparation of Electrolyte

The preparation procedure for the electrolyte solution of 0.25 mol L^−1^ Mg(AlCl_2_EtBu)_2_/THF was previously reported in detail [16]. Briefly, MgBu_2_ solution (1 mol L^−1^ in hexane, Aldrich) and AlCl_2_Et solution (0.9 mol L^−1^ in heptane, Aldrich) were mixed at a 1 : 2 ratio at room temperature, which caused a white solid to immediately precipitate. After stirring for 48 h, the solvents (hexane and heptane) were removed by evaporation, and high purity THF (distilled with benzophenone containing sodium chips under an Ar atmosphere) was added to form the desired 0.25 mol L^−1^ solution. All the chemical preparations were performed in an Ar-filled glove box (Mbraun, Unilab, Germany).

### 2.3. Structural and Morphological Characterization

X-ray powder diffraction (XRD) analysis was performed using a Rigaku D/MAX-2200/PC X-Ray Diffractometer (Japan) equipped with a Cu K*α* radiation source (*λ* = 0.15406 nm) to analyze the components of the products. Solid-state ^13^C NMR was performed with an Avance III 400 spectrometer (Bruker Inc., Switzerland). Raman spectrum was carried out on a Dispersive Raman Microscope (Senterra R200-L, Bruker Optics, Germany). Thermogravimetric (TG) analysis was performed at a Thermogravimetric Analyzer (TGA 7, Perkin Elmer, Inc., USA) and differential scanning calorimeter (DSC) analysis was performed at a Differential Scanning Calorimeter (Pyris 1, Perkin Elmer, Inc., USA). The elements of the material were analyzed by an inductively coupled plasma-atomic emission spectroscopy (ICP-AES) using an Iris Advantage 1000 spectroscope (Thermo Electron). The microscopic features of the product were observed using scanning electron microscopy (SEM) on a JEOL field-emission microscope (JSM-7401F) and transmission electron microscopy (TEM) on a JEOL high-resolution electron microscope (JEM-2010). N_2_ adsorption-desorption experiments were carried out at −196°C on a ASAP 2010 M+C surface area and pore analyzer (Micromeritics, USA) after degassing of the samples at 200°C for 3 hours. The specific surface area of sample was determined by Brunauere-Emmette-Teller (BET) measurement with nitrogen gas on an ASAP 2010 M+C surface area analyzer (Micromeritics, USA).

### 2.4. Electrochemical Measurements

75 wt.% carbyne polysulfide active material, 15 wt.% super-P carbon powder, and 10 wt.% poly(vinylidene fluoride) (PVDF) dissolved in N-methyl-2-pyrrolidinone (NMP) were mixed and coated onto a copper foil with a typical thickness of 50 *μ*m. After volatilization of the solvent, the coated current collector was punched into 12 mm diameter disks, pressed at 3 MPa, and dried at 80°C for 10 h under vacuum to form electrode disks loaded with active materials. Cyclic voltammetry (CV) measurement was conducted via a three-electrode cell on a CHI650C electrochemical workstation (CH Instruments Inc., USA). The working electrode had the same composition as mentioned above, magnesium ribbon (1 mm diameter) (Aldrich) acted as both the reference and counter electrode, and 0.25 mol L^−1^ Mg(AlCl_2_EtBu)_2_/THF as the electrolyte. The electrochemical performance of the active material was further examined via CR2016 coin cells with a magnesium ribbon counter electrode, an Entek PE membrane separator. The three-electrode cells and coin cells were assembled in an Ar-filled glove box. Galvanostatic charge-discharge measurements were conducted on a Land battery measurement system (Wuhan, China) with a cut-off voltage between 1.8 V and 0.5 V versus Mg. All the electrochemical tests were performed at room temperature.

## 3. Results and Discussion

The element analysis of products (S1, S2, S3, S4, and S5) prepared by coheating carbyne and sulfur at different temperatures is shown in Table 1. The main elements are S and C with few H and Cl. The molar ratio of S : C for S1, S2, and S3 is over 5 : 1, which is mainly resulted from little loss of elemental sulfur lower than 250°C and the consumption of some carbon with oxygen-containing groups (produced from the reaction of PVDC and KOH) in the products. The ratio of S/C decreases to 1.28 : 1 for S4 and 0.59 : 1 for S5 with the increase of heating temperature, indicating the sublimation of most of elemental sulfur at the temperature higher than 300°C.

**Figure 1:**
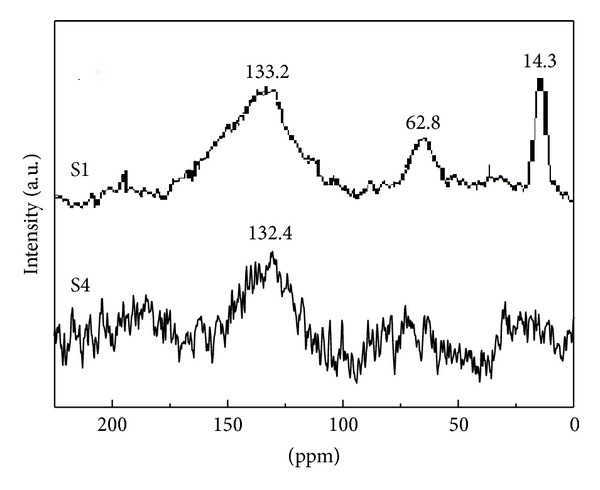
^13^C-NMR spectra of S1 and S4.

Figure 1 shows the solid-state ^13^C NMR of S1 and S4. There is a peak at 133.2 and 132.4 ppm for S1 and S4, respectively. It corresponds to the sp^2^ hybridization of carbon, suggesting that both S1 and S4 contain carbon-carbon double bonds. For S1, two additional peaks at 14.3 ppm and 62.8 ppm are related to –CH_3_ and sp hybridization of carbon, respectively [17]. It is demonstrated that the product prepared at 150°C exhibits a compound structure with triple bonds, double bonds, and –CH_3_ groups resulting from side reaction. The single peak for S4 illustrates that the product prepared at 300°C has a single structure.

**Figure 2:**
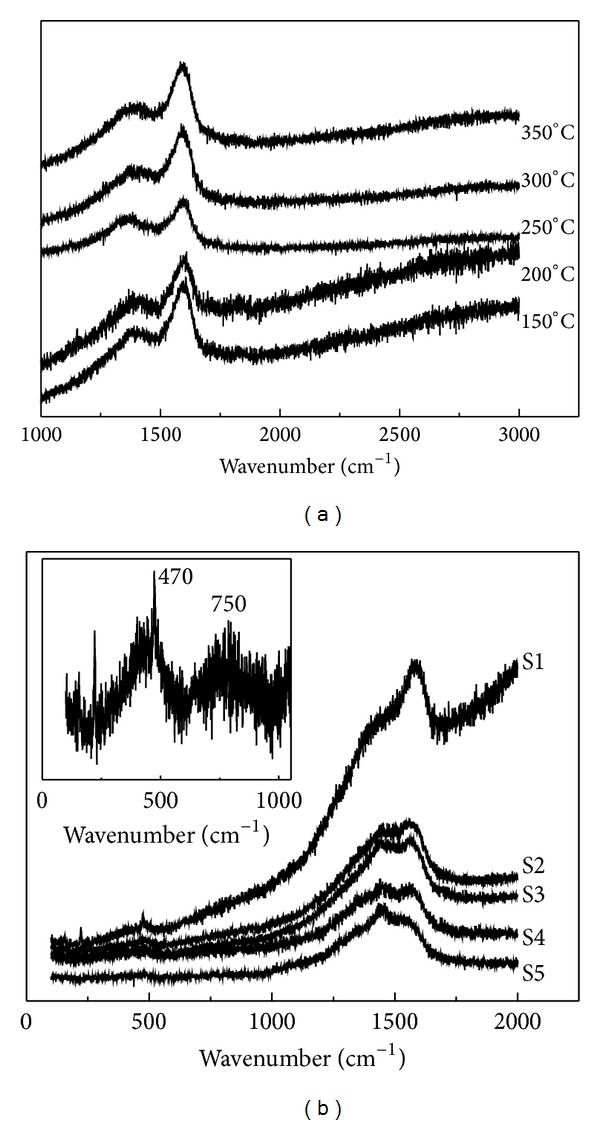
Raman spectrum of (a) carbyne treated at different temperatures, (b) S1, S2, S3, S4, and S5; inset is a partial magnification for the spectrum of S4.

**Figure 3:**
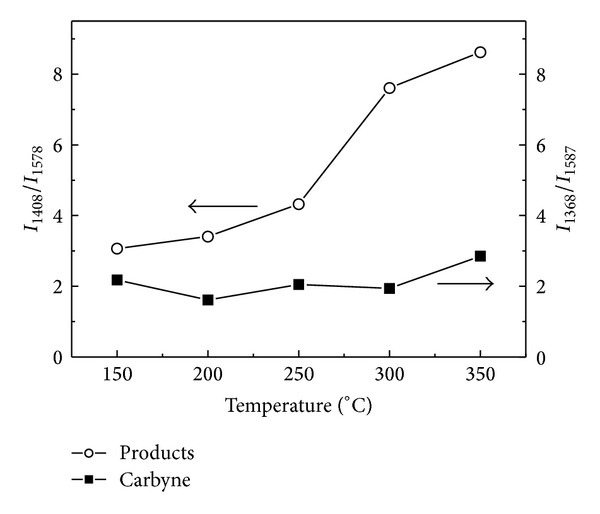
The intensity ratio between vibration modes of products and carbyne treated at different temperatures.

Raman spectrum was further employed to confirm the structure of the products. Figures 2(a) and 2(b) show the Raman spectra of carbyne, which was heat-treated at different temperatures, and the products prepared by coheating carbyne and sulfur at different temperatures. Two peaks appear at about 1368 cm^−1^ and 1587 cm^−1^ for carbyne and 1408 cm^−1^ and 1578 cm^−1^ for the products. The variation of the ratio of the two peaks with temperature is shown in Figure 3. Compared with that for carbyne, the value for the products increases obviously with the increasing temperature. As a result, the difference of carbon structure between carbyne and the products does not result from the heat treating temperature but the interaction of carbyne with sulfur during the heat treating process. It was reported that the peaks at 1580 cm^−1^ and 1360 cm^−1^ can be attributed to the carbon vibration of graphite crystallite at the centers and edges, which were called G and D vibration modes, respectively [18]. The peak at 1368 cm^−1^ for carbyne shifts to 1408 cm^−1^ after coheating treatment of carbyne and sulfur. It is suggested that the carbon vibration of graphite crystallite at edges is influenced by sulfur. A chemical reaction not the simple mixture occurs between sulfur and carbyne. *I*_1408_/*I*_1578_ increases with the coheating temperature, indicating that the carbon at edges reacts with more sulfur at high temperature. As shown in inset of Figure 2(b), a peak with low intensity appears at 750 cm^−1^. It can be attributed to C–S bonds [19]. For S1, S2, S3, and S4, there is a peak appearing at 470 cm^−1^, suggesting the existence of S–S bonds. The peak disappears in the spectrum of S5. It is demonstrated that the S–S bonds do not exist in the product when the coheating temperature reaches 350°C.

**Figure 4:**
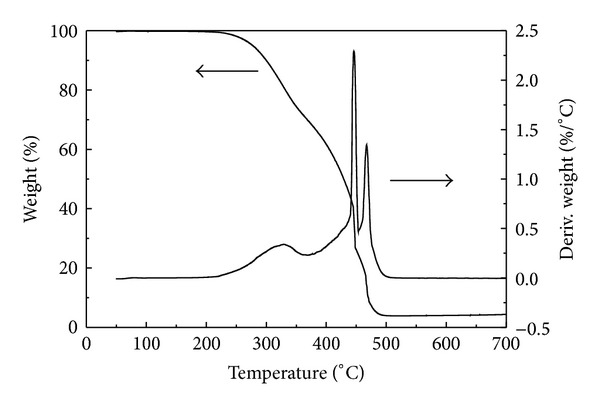
TG analysis of S4.

**Figure 5:**
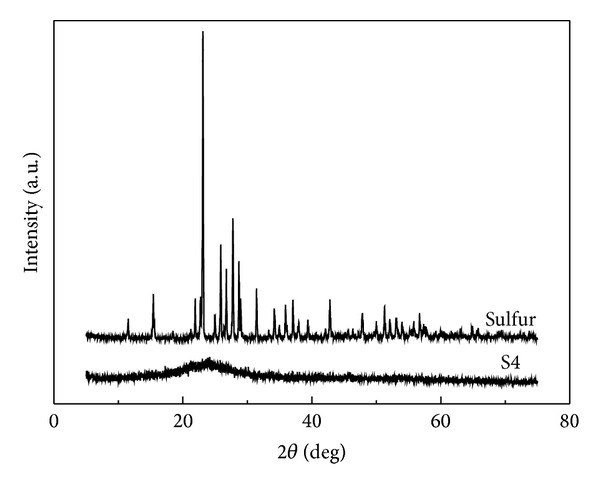
XRD patterns of element sulfur and S4.

The results of TG/DSC (not shown) for the products demonstrate that there are four endothermic peaks at 102.0, 119.3, 167.0, and 333.8°C for S1, S2, and S3. The former three and the last are related to crystal phase transition in melting and sublimation of elemental sulfur, respectively. The peaks become weaker for S4 and unobvious for S5. TG analysis of S4 in Figure 4 shows a low weight loss at about 330°C, suggesting a small amount of elemental sulfur in the material. There is about 60% and 30% weight loss at above 400°C for S4 and S5, respectively, indicative of a moderate thermal stability due to the chemical combination between sulfur and carbon. XRD pattern shown in Figure 5 further demonstrates that S4 mainly contains amorphous carbon with a preferred orientation at (002) lattice plane.

From what has been discussed above, it can be concluded that there is no chemical combination between sulfur and carbon at the coheating temperature lower than 250°C. When the temperature increases to 300°C, most of sulfur links in the form of S–S bonds to the carbon matrix with sp^2^ hybridization. The structure is favorable to provide a conducting carbon skeleton and energy-storing sulfur side chain. However, S–S bonds decompose when the temperature reaches 350°C. The product prepared at 300°C (called carbyne polysulfide) was chosen for further electrochemical measurements.

**Figure 6:**
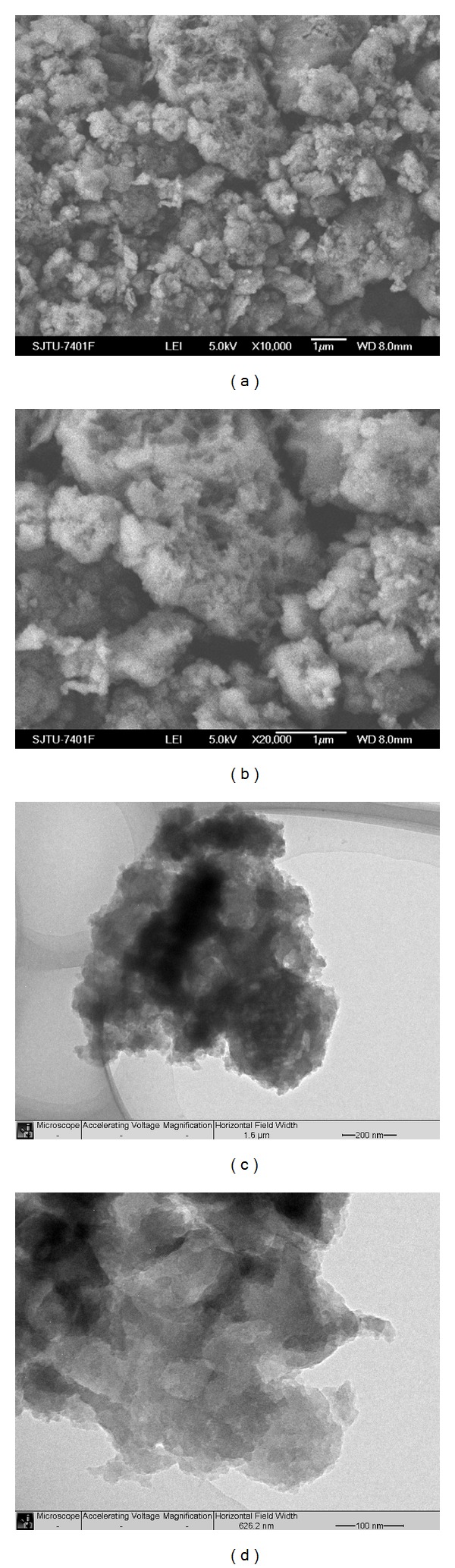
SEM (a and b) and TEM (c and d) images of carbyne polysulfide.

Figures 6(a) and 6(b) show SEM images of carbyne polysulfide with different magnification. The material features nonuniform particles ranged from hundreds of nanometers to a few micrometers. A closer observation (Figure 6(b)) shows that some particles are composed of porous structures. The morphology and microstructure of the material were further investigated by TEM. Figures 6(c) and 6(d) show the typical images with different magnification. The formation of a porous framework with different sizes of pores is clearly observed. In this structure, the electrolyte will effectively fill the particles. The porous structure is probably due to the release of large quantity of sulfur vapor during the sintering process.

**Figure 7:**
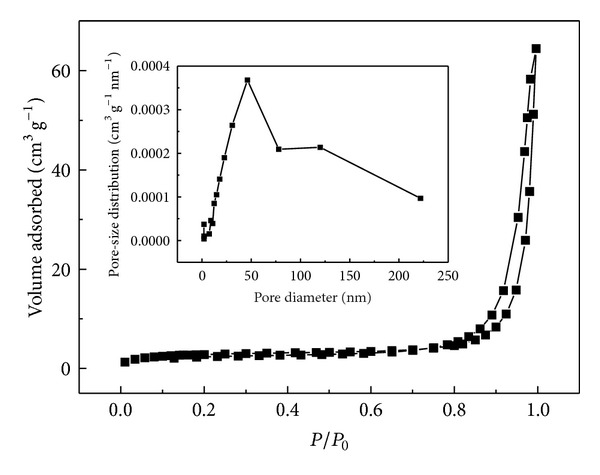
Nitrogen adsorption-desorption isotherms of carbyne polysulfide. The inset shows the pore-size distribution.

The porosity of carbyne polysulfide can be further confirmed by nitrogen adsorption-desorption analysis. The nitrogen adsorption-desorption isotherm for carbyne polysulfide is shown in Figure 7. The isotherm is of type II classification with a hysteresis. Type II isotherms are characteristic of multilayer adsorption on nonporous or macroporous solids [20]. Porosity characteristics are more evidently observed from BJH curve (the inset of Figure 7), which reflects the presence of pores of two different sizes. There is a narrow pore-size distribution at approximately 50 nm and a broad distribution of pores around 120 nm. The specific surface area calculated by the Brunauer-Emmett-Teller (BET) method and the pore volume determined by the Barrett-Joyner-Halenda (BJH) approach are 9.7624 m^2 ^g^−1^ and 0.099687 cm^3 ^g^−1^, respectively.

**Figure 8:**
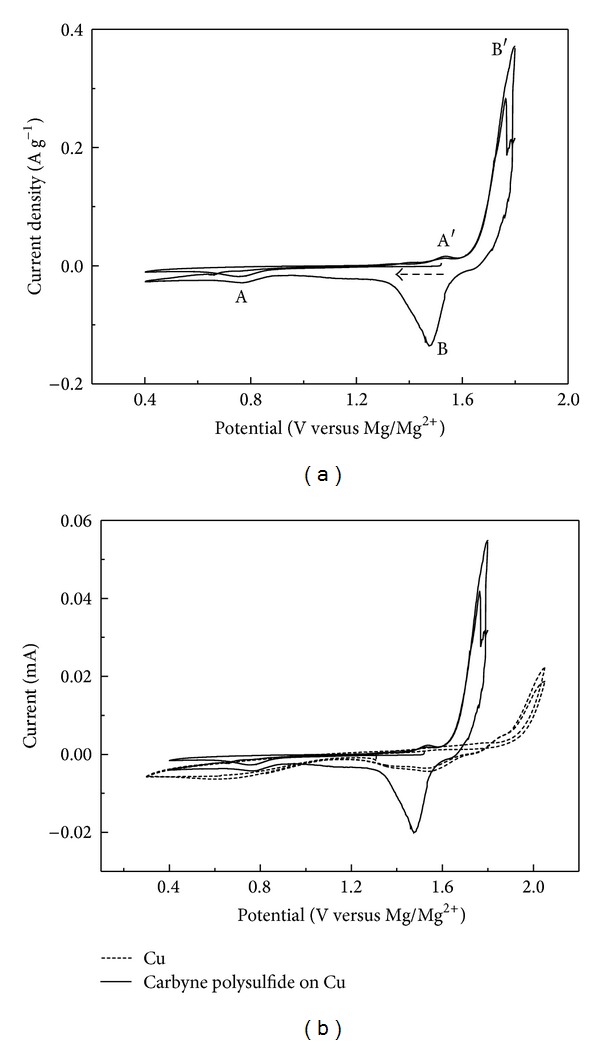
(a) Cyclic voltammograms of carbyne polysulfide in a three-electrode cell employing magnesium metal as the counter electrode and the reference electrode measured at a scan rate of 0.05 mV s^−1^. (b) CV comparisons using Cu copper collector and carbyne polysulfide on copper collector as positive electrodes. The active mass of carbyne polysulfide was 0.1476 mg.

Cyclic voltammogram (CV) and galvanostatic discharge-charge techniques were used to investigate the electrochemical behavior of the carbyne polysulfide material for electrochemical magnesium storage.  Figure 8(a) shows CV curves of carbyne polysulfide in a three-electrode cell employing magnesium metal as the counter electrode and the reference electrode at a scan rate of 0.05 mV s^−1^. The voltammograms demonstrate two pairs of cathodic/anodic peaks, which are marked as A/A′ and B/B′, respectively. The process can be attributed to the electrochemical reaction of a small amount of elemental sulfur with magnesium and the cleavage and linkage of S–S bonds, respectively. In view of sulfur mainly in the form of S–S bonds in the material, the cleavage and recombination of S–S bonds formed during reduction and oxidation process is the key components for the electrochemical reaction. It has been reported that the copper current collector can react with Mg(AlCl_2_EtBu)_2_/THF electrolyte above 1.8 V [21]. In the CV measurements, the anodic current was observed beginning from 1.8 V and there was an oxidation peak at 2.05 V, which was ascribed to the electrochemical oxidation of Cu. A reduction peak was observed at about 1.0 V, which may be ascribed to electrochemical reduction of Cu^2+^ or Cu^+^ to Cu. Considering the possible disturbance of copper collector, the CVs comparison of Cu substrate and carbyne polysulfide active material on copper collector is shown in Figure 8(b). There are indeed oxidation and reduction processes related to the reaction of copper current collector with the electrolyte when the upper potential limit is over 1.8 V. However, the electrochemical process below 1.8 V should be related to the reaction of the active material with magnesium.

**Figure 9:**
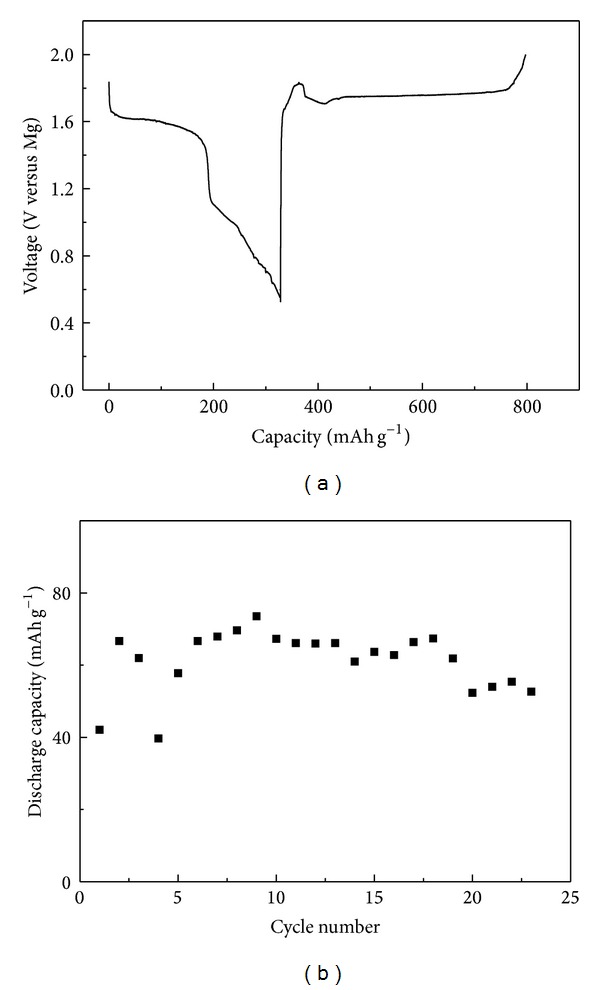
(a) A typical discharge-charge curve of carbyne polysulfide in a two-electrode coin cell employing magnesium metal as the counter electrode at a current rate of 3.9 mA g^−1^. (b) The discharge capacity versus cycle number of carbyne polysulfide in a two-electrode coin cell employing magnesium metal as the counter electrode at a current rate of 5.4 mA g^−1^.

Figure 9(a) shows a typical discharge and charge curve of carbyne polysulfide in a two-electrode coin cell employing magnesium metal as the counter electrode at a current rate of 3.9 mA g^−1^. The discharge curve demonstrates two separate plateaus of 1.6 V and 1.1 V related to the reduction peaks of B and A in Figure 8(a). The discharge corresponds to 327.7 mAh g^−1^ capacity, that is, 53.8% of the theoretical value 609.1 mAh g^−1^. The high capacity may be resulted from the special structure of carbyne polysulfide with a conducting carbon skeleton and energy-storing sulfur side chain without other elements. The discharge capacity versus cycle number of carbyne polysulfide at a higher rate of 5.4 mA g^−1^ is shown in Figure 9(b). The material exhibits an activation process during the initial cycles due to the gradual infiltration of electrolyte into the electrode. The slow penetration of electrolyte into the pores and some parasitic reactions occurring on the material also result in an unstable capacity upon cycling. As shown in the figure, the discharge capacity and the cycling performance are not satisfied. It has been concluded that carbyne polysulfide contains a conducting carbon skeleton and energy-storing sulfur side chain, which should keep the discharge products remaining in the skeleton during the cycling process. Further work is aimed at choosing a more matchable electrolyte system to improve the capacity retention of carbyne polysulfide.

## 4. Conclusions 

Carbyne polysulfide material was prepared by coheating carbon containing carbyne moieties and elemental sulfur. The product was proved to have a sp^2^ hybrid carbon skeleton with polysulfide attached on it. The structure is favorable to provide a conducting carbon skeleton and energy-storing sulfur side chain. The electrochemical measurements exhibited that carbyne polysulfide can be used as a novel cathode material and the material has a high capacity of 327.7 mAh g^−1^ at a current rate of 3.9 mA g^−1^.

## Figures and Tables

**Table 1:** Elemental analysis result of the products prepared by co-heating carbyne and sulfur at different temperatures.

Sample	Temperature/°C	Element content/%				Composition
		C	H	Cl	S	
S1	150	6.23	0.64	0.74	91.51	CH_1.21_Cl_0.04_S_5.40_
S2	200	5.91	0.77	0.75	92.41	CH_1.57_Cl_0.04_S_5.89_
S3	250	6.47	1.11	0.51	91.60	CH_2.06_Cl_0.03_S_5.30_
S4	300	21.94	0.94	1.15	74.49	CH_0.52_Cl_0.02_S_1.28_
S5	350	36.10	0.87	2.09	57.13	CH_0.29_Cl_0.02_S_0.59_
